# Prevalence of Hyperuricemia and Its Association with Cardiovascular Risk Factors and Subclinical Target Organ Damage

**DOI:** 10.3390/jcm12010050

**Published:** 2022-12-21

**Authors:** Paula Antelo-Pais, Miguel Ángel Prieto-Díaz, Rafael M. Micó-Pérez, Vicente Pallarés-Carratalá, Sonsoles Velilla-Zancada, José Polo-García, Alfonso Barquilla-García, Leovigildo Ginel-Mendoza, Antonio Segura-Fragoso, Facundo Vitelli-Storelli, Vicente Martín-Sánchez, Álvaro Hermida-Ameijerias, Sergio Cinza-Sanjurjo

**Affiliations:** 1 Santa Comba Health Centre, Health Area of Santiago de Compostela, 15970 Santiago de Compostela, Spain; 2 Vallobín-La Florida Health Centre, 33012 Oviedo, Spain; 3 Fontanars dels Alforins Health Centre, Xàtiva–Ontinyent Department of Health, 46635 Valencia, Spain; 4 Health Surveillance Unit, Mutual Insurance Union, 12004 Castellon, Spain; 5 Department of Medicine, Jaume I University, 12071 Castellon, Spain; 6 Joaquin Elizalde Health Centre, 26004 Logroño, Spain; 7 Casar de Cáceres Health Centre, 10200 Cáceres, Spain; 8 Trujillo Health Center, 10200 Cáceres, Spain; 9 Ciudad Jardín Health Center, 29014 Málaga, Spain; 10 Epidemiology Unit, Semergen Research Agency, 28029 Madrid, Spain; 11 Gene-Environment-Health Interaction Research Group (GIIGAS)/Institute of Biomedicine (IBIOMED), University of León, 24071 León, Spain; 12 Institute of Biomedicine (IBIOMED), Epidemiology and Public Health Networking Biomedical Research Centre (CIBERESP), University of León, 24071 León, Spain; 13 Department of Internal Medicine, University Hospital of Santiago de Compostela, 15706 A Coruña, Spain; 14 Porto do Son Health Centre, Health Area of Santiago de Compostela, Health Research Institute of Santiago de Compostela (IDIS), Networking Biomedical Research Centre-Cardiovascular Diseases (CIBERCV), 15970 Santiago de Compostela, Spain

**Keywords:** hyperuricemia, cardiovascular risk, prevalence, associated risk

## Abstract

The role of uric acid levels in the cardiovascular continuum is not clear. Our objective is to analyze the prevalence of hyperuricemia (HU) and its association with cardiovascular risk factors (CVRF), subclinical target organ damage (sTOD), and cardiovascular diseases (CVD). We evaluated the prevalence of HU in 6.927 patients included in the baseline visit of the IBERICAN study. HU was defined as uric acid levels above 6 mg/dL in women, and 7 mg/dL in men. Using adjusted logistic regression models, the odds ratios were estimated according to CVRF, sTOD, and CVD. The prevalence of HU was 16.3%. The risk of HU was higher in patients with pathological glomerular filtration rate (aOR: 2.92), heart failure (HF) (aOR: 1.91), abdominal obesity (aOR: 1.80), hypertension (HTN) (aOR: 1.65), use of thiazides (aOR: 1.54), left ventricular hypertrophy (LVH) (aOR: 1.36), atrial fibrillation (AFIB) (aOR: 1.29), and albuminuria (aOR: 1.27). On the other hand, being female (aOR: 0.82) showed a reduced risk. The prevalence of HU was higher in men, in patients presenting CVRF such as HTN and abdominal obesity, and with co-existence of LVH, atrial fibrillation (AFIB), HF, and any form of kidney injury. These associations raise the possibility that HU forms part of the early stages of the cardiovascular continuum. This may influence its management in Primary Healthcare because the presence of HU could mean an increased CV risk in the patients.

## 1. Introduction

Cardiovascular diseases (CVD) are the leading cause of death in Spain, particularly in women [1], and are also the second leading cause of years of life lost after cancer [2]. A decreasing trend, in standardized rates, has been observed in the last few decades which is due to the improvements in primary prevention, lifestyle modification, better control of cardiovascular risk factors (CVRF), and better management and treatment, mainly interventional, in the acute phase of events [3]. However, despite the better control of CVRF, many patients develop CVD, suggesting that there may be remaining factors which are not controlled.

Atherosclerosis is the pathophysiological base of CVD, which progresses silently over decades due to the effect of CVRF and of subclinical target organ damages (sTOD) to the development of CVD [4]. This is what is known as the cardiovascular continuum.

Among CVRF, the potential role of uric acid—a product of xanthine oxidase—in the development of CVD has been set forth, since high uric acid levels have been identified in the context of oxidative stress and of CVD, such as ischemic heart disease (IHD) and heart failure (HF) [5].

Hyperuricemia (HU) is associated with a state of oxidative stress that produces endothelial dysfunction, increases oxidation of LDL, and promotes a proinflammatory state which contributes to the development of atherosclerosis and its thrombotic complications [6,7,8,9]. The presence of uric acid in the atheromatous plaque is the reason why the classical cut-off points defining HU (6 mg/dL in women, and 7 mg/dL in men) are based on the levels of precipitation in uric acid [10,11].

At present, there are studies available that provide solid results on the relationship between HU and CVRF such as hypertension (HTN) [12], diabetes mellitus (DM) [13], obesity [14], hypercholesterolemia [15], and metabolic syndrome (MetS) [13,16], as well as between HU and sTOD [17,18]. Nevertheless, results are more controversial and contradictory regarding its association with CVD [19,20,21]. These discrepancies could be justified by the presence of uncontrolled confounding factors in those studies, for example, the difference between the sexes, the peripheral insulin resistance, or the MetS [22]. The real role of HU and how it is associated with other CVRF has remained unknown until now. A relationship between HU and several metabolic conditions (DM, MetS, obesity, or HTN) has been suggested. In addition, some authors explain the association between HU and a worse prognosis in patients with an acute coronary disease [23] or after the initial hospitalization for HF [24].

Forming a complete picture about patients and the CVRF, the risk factors needing to be controlled, and the sTOD or CVD rates would allow us to control the confounding effect among all the variables and to further the study of the role of HU in the cardiovascular continuum, as is the case of the IBERICAN study (Identification of Spanish Population in Cardiovascular and Renal Risk) [25].

The objective of this study is to analyze which CVRF, sTOD, and CVD are associated with HU in patients seen in primary care practices.

## 2. Materials and Methods

### 2.1. Study Design

Cross-sectional HU prevalence study conducted in patients included in the IBERICAN study [25].

The study was approved by the Ethics Committee for the investigation with medicinal products (CEIm) of the Hospital Clínico San Carlos of Madrid on 21 February 2013 (C.P. IBERICAN-C.I. 13/047-E) and is registered at https://clinicaltrials.gov with the number NCT02261441 (accessed on 1 November 2022).

### 2.2. Selection of Patients

Patients aged between 18 and 85 were recruited consecutively in primary care practices with the following inclusion criteria: (1) user of the National Health System, (2) residing in Spain during the last 5 years, (3) registered with the physician researcher, (4) signing the informed consent form. The exclusion criteria were (1) change of habitual residence to another town or country in the next 6 months, (2) terminal illness or reduced life expectancy in the next 5 years, (3) clear difficulty in being followed up in PC, or (4) refusal to form part of the cohort in the first place or to continue to be part of it during the follow-up. The estimated sample size has been detailed in previous publications [25].

### 2.3. Variables Recorded

Socio-demographic data of each patient were recorded in the entry visit (sex, age, habitat, level of education, family economic status, and current employment situation), as well as toxic habits (tobacco and alcohol consumption), family history of early CVD and personal history (HTN, DM, hypercholesterolemia, atrial fibrillation—AFIB-, HF, peripheral artery disease, or cerebrovascular disease), clinical parameters (weight, height, body mass index—BMI-, waist circumference, systolic pressure, diastolic pressure, pulse pressure—PP-, and heart rate), information on the presence or absence of each CVRF as well as their treatment. The complementary tests recorded were lab tests (blood count, blood chemistry, and urine test) and electrocardiogram, which were considered valid if performed within the last 6 months before the patient’s inclusion. The equation CKD-EPI was used to calculate the glomerular filtration rate (eGFR) [26].

The HU was defined as uric acid levels above 6 mg/dL in women and 7 mg/dL in men, as set out in the guidelines on HTN of the European societies 2018 [27]. All other variables have been described in previous publications [25], and the CVRF together with their degree of control have been defined according to the respective clinical practice guidelines [21]. The term CVD includes here IHD, HF, peripheral artery disease, and cerebrovascular disease, as set out in the clinical practice guidelines [27].

### 2.4. Statistical Analysis

Qualitative variables have been defined as percentages with a 95% confidence interval (95% CI). For quantitative variables, the Shapiro–Wilk test has been used to check the data fitting to normal distribution. If the variable showed normal distribution, it has been described using the arithmetic mean and the standard deviation (SD); otherwise, the median and the interquartile range have been used.

The bivariate analysis has been performed using chi-square for qualitative variables, and ANOVA for quantitative variables, comparing patients with HU with patients without HU.

Unconditional logistic regression models were used to estimate the odds ratios adjusting (aOR), as the case may be, for socio-demographic variables (sex, age, level of education, habitat, employment, income, and race) or clinical variables. Results are presented with their 95% confidence intervals (95% CI). Finally, a multivariate analysis was carried out to study the relationship of CVRF and demographic variables with HU. All analyses were performed with the program STATA version 16. We consider statistically significant a *p*-value < 0.05.

## 3. Results

The study included the 6927 patients with available data on uric acid levels. The prevalence of HU was 16.3% (95% CI: 15.4–17.1). Table 1 shows the prevalence of HU according to the patients’ socio-demographic characteristics. The risk of developing HU was lower in women (aOR: 0.73 [95% CI: 0.64–0.84]) and increased with age (aOR: 1.02 [95% CI: 1.02–1.03]). The risk of HU was lower in rural habitats as against urban habitats (aOR: 0.82 [95% CI: 0.68–0.97]), and in patients with primary education (aOR: 0.80 [95% CI: 0.64–0.99]) and with higher education (aOR: 0.70 [95% CI: 0.51–0.96]) as against patients with lower educational level.

In total, 35.4% (95% CI: 32.6–38.3%) of patients with HU presented some form of sTOD. The sTOD which had the highest risk of HU was left ventricular hypertrophy (LVH) (aOR: 1.36 [95% CI: 1.00–1.83]) (Table 2).

A total of 23.9% of patients with HU presented CVD. A significantly higher prevalence of HU was observed in patients with AFIB (aOR: 1.53 [95% CI: 1.19–1.98]) (Table 2). The risk of HU was also higher in patients with kidney injuries, both in eGFR < 60 mL/min (aOR: 3.01 [95% CI: 2.40–3.78]) and in albuminuria (aOR: 1.43 [95% CI: 1.14–1.78]).

**Figure 1:**
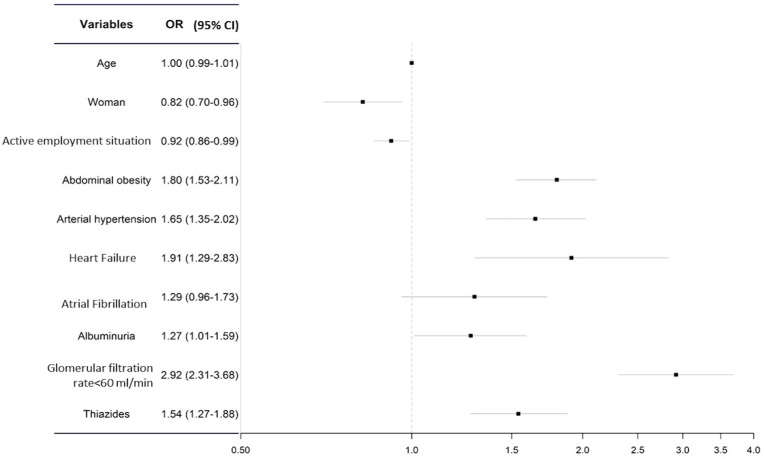
Multivariate analysis for the risk of hyperuricemia.

The model best-fitting model (Figure 1) includes the following variables: age and sex, abdominal obesity, HTN, HF, AFIB, albuminuria, glomerular filtration rate < 60 mL/min, and use of thiazides. It can be seen that a pathological glomerular filtration, HF, abdominal obesity, HTN, use of thiazides, and albuminuria were associated with a higher risk of HU. On the other hand, being female and being actively employed were the only variables which were found to be protective factors.

## 4. Discussion

The results presented here, from a sample of 6927 patients recruited consecutively in PC, show that the prevalence of HU is 16.3%, and that the risk of HU increases in patients with both forms of kidney disease (albuminuria and glomerular filtration rate < 60 mL/min), HF, AFIB, and in the presence of CVRF such as HTN and abdominal obesity, as well as the use of thiazides. These associations have been described in various studies independently, although this is the first time that all the clinical and epidemiological variables are analyzed at the same time in a contemporary PC cohort. This has allowed us to analyze the risk of HU associated simultaneously with other variables, in order to be able to make the necessary adjustments in a multiple analysis.

The HU prevalence (16.3%) found in this study is slightly higher than other works that used the same cut-off points in neighbouring countries (13.9%) [28], or even in Asian cohorts (13.2% and 13.5%) [29,30]. Our sample shows a higher prevalence because it is a clinical practice sample, with a higher prevalence of CVRF and CVD than other population samples, as these three studies. In other ways, compared to these studies, we observed a higher prevalence in males and an increasing rate with age. This is usually because the uricemia levels increase in men aged 20 to 65 and in women older than 60; this explains that the prevalence of HU is higher in men when we analyze younger samples, and higher in women when we analyze older samples [31]. In view of this, we should consider whether, in order to define the HU as a risk factor, different cut-off points should be used not only by sex but also by age groups for both sexes. HU could be a secondary association with insulin resistance, dyslipidemia, and obesity, which are associated with an inflammatory condition state. However, uric acid is part of the body’s antioxidant defences, and is still questioned if higher levels of uric acid are a protective response to damage or a cause of disease.

Our results, obtained from a cross-sectional analysis of the entry visit of the patients of the IBERICAN study, do not enable us to establish the causal link of these CVRF and HU. Nevertheless, these results reinforce the association with obesity [32], HTN [12,33], DM [34,35], MetS [36], and even a more atherogenic lipid profile [37]. After a slow reading of these results, it is not clear if the HU is a consequence of obesity and after favours the development of HTN, hypercholesterolemia, and DM [38]. This could be related to an increased activity of xanthine oxidase in obese patients [39], but there is not enough evidence in the literature to answer this question.

This study also allowed us to analyze the simultaneous coexistence of sTOD with CVRF and CVD. It has been observed that both albuminuria and LVH have a higher risk of associated HU. This is important because both lesions are closely linked with HTN [33,40,41], but also in patients with metabolic disorders such as obesity and DM [42,43]. Something similar happens to those CVD cases associated with a higher risk of HU, such as AFIB and HF, both directly related to HTN [44]. HU was also associated with a worse prognosis in patients after acute coronary disease [23,45]. Finally, the relationship between kidney disease and HU has been confirmed, which has already been described by other authors [46,47]. This relationship has a two-way effect between the two of them [48], where both the activation of inflammation [49] and the activation of the renin–angiotensin system [50] may be involved. All this evidence might reinforce the hypothesis stated above on the role of HU in the cardiovascular continuum, from the CVRF to CVD: HU is an early marker of CVR closely linked to obesity, since the pro-inflammatory role of uric acid has been described [51], and is considered one of the pathophysiological bases of atherosclerosis [52].

From a practical point of view, HU appears to be a risk marker that reflects the oxidative stress associated with a higher CVR, from the first stages to recurrent events, and the development of kidney disease and CVD. In the absence of treatments that improve the CV prognosis with the reduction of uric acid levels, identifying patients with higher levels of uric acid could be useful to identify individuals with a higher associated CVR, and who could benefit from a better control of CVRF, giving priority to obesity, in order to improve the CV prognosis. In parallel, a careful use of thiazides should be considered in patients with HTN, since the results of our multivariate analysis have shown that both affect the risk of HU. So, taking into account that it is a first-line drug for HTN [27] and a known cause of HU [8], and a known cause of HU, the avoidance of taking thiazides seems to be a reasonable strategy for preventing HU.

### Strengths and Limitations

We believe that the results presented here are statistically robust and consistent with the available literature on this topic; in addition, they allow for a better understanding of the characteristics of the patients with HU in a contemporary cohort. The sample size is sufficient to analyze which CVRF are associated with HU, as well as the adjustment variables necessary for this study. However, our research also has limitations, some of which have been discussed in previous works [25,53], such as the voluntary participation of the investigators or the use of physical examination devices available in the practice with no regular calibration. In this sense, the information obtained in our analysis may not represent the global prevalence of HU in our country, but our main objective was to analyze the relationship between HU and other clinical situations related to cardiovascular risk including CVRF, TOD, and CVD. Despite these limitations, the authors consider it not to have influenced our findings.

Moreover, the cross-sectional design of the study does not allow us to establish causal relationships, although the aim of this study was to describe the patients’ characteristics and, therefore, its design is valid.

Lastly, in this research, the uric acid-lowering treatment of the patients was not recorded; therefore, it could not be included in the adjustment models. In this sense, it is possible that some patients classified as normouricemic would actually be HU. Even keeping that in mind, our results show the best situation, and by avoiding this bias, we would obtain more strength associations as we observed. Moreover, our results are similar to those of other authors published before, and they are biologically plausible. For these reasons, authors consider our findings answer our main research question.

## 5. Conclusions

Given the foregoing findings, it can be concluded that the risk of HU is greater in men, in the presence of CVRF such as HTN and abdominal obesity, and with the coexistence of LVH, AFIB, HF, and any form of kidney injury. These associations raise the possibility that the HU forms part of the early stages of the cardiovascular continuum, but it needs observational studies that confirm this hypothesis. However, it remains to be seen whether it is a cardiovascular risk factor and a possible therapeutic target to modify the CV prognosis, or a reflection of the existence of other metabolic disorders and of the oxidative stress characteristic of abdominal obesity which are mainly responsible for increasing the CV risk in this group of patients. If we can confirm this association, its presence in the early phases of the cardiovascular continuum makes it necessary to pay more attention in primary care to patients classified as low or moderate cardiovascular risk who have increased uric acid levels, since it could lead to reassessment of CVR.

## Figures and Tables

**Table 1:** Distribution in the prevalence of hyperuricemia according to various socio-demographic variables.

Variable		N	n	%	OR 95% CI	aOR 95% CI
Sex	Man	3188	597	18.7	0.72 [0.63–0.81]	0.73 [0.64–0.84]
	Female	3739	529	14.1		
Race	White	6678	1097	16.4		
	Others	249	29	11.6	0.67 [0.45–0.99]	0.78 [0.52–1.16]
Habitat	Urban	4061	683	16.8		
	Semi-urban	1474	237	16.1	0.95 [0.81–1.11]	0.97 [0.82–1.15]
	Rural	1390	206	14.8	0.86 [0.73–1.02]	0.82 [0.68–0.97]
Education	Uneducated	627	136	21.7		
	Primary	3814	622	16.3	0.70 [0.57–0.87]	0.80 [0.64–0.99]
	Secondary	1561	251	16.1	0.69 [0.55–0.87]	0.88 [0.67–1.14]
	Tertiary	925	117	12.6	0.52 [0.40–0.69]	0.70 [0.51–0.96]
Employment	Employed	2914	404	13.9		
	Unemployed	569	69	12.1	0.86 [0.65–1.13]	0.88 [0.66–1.17]
	Retired	2485	514	20.7	1.62 [1.04–1.87]	0.93 [0.76–1.14]
	Student	76	6	7.9	0.53 [0.23–1.23]	0.95 [0.40–2.24]
	Domestic chores	861	131	15.2	1.11 [0.90–1.38]	0.92 [0.71–1.18]
Salary (EUR/year)	<18,000	2920	490	16.8		
	≥18,000	4007	636	15.9	0.94 [0.82–1.06]	1.01 [0.87–1.16]
Age, mean (SD)	HU	62.7 (13.3)			1.02 [1.02–1.03]	1.02 [1.02–1.03]
	No HU	57.9 (14.7)				

OR = odds ratio; 95% CI = 95% confidence interval; aOR = odds ratio adjusted for sex, race, habitat, education level, employment, salary, and age. HU = hyperuricemia. Age is presented as mean and standard deviation (SD).

**Table 2:** Distribution in the prevalence of hyperuricemia according to cardiovascular risk factors, target organ damage, and history of cardiovascular disease.

Variable			N	n	%	OR 95% CI	aOR 95% CI
CVRF	Abdominal Obesity	No	4691	573	12.2	2.25 [1.97–2.58]	1.56 [1.09–2.24]
		Yes	2074	513	24.7		
	Obesity	No	4445	531	11.9	2.20 [1.92–2.52]	1.29 [0.90–1.84]
		Yes	2320	555	23.9		
	HTN	No	3469	340	9.8	2.31 [1.97–2.70]	1.95 [1.66–2.30]
		Yes	3292	746	22.7		
	Hypercholesterolemia	No	3319	422	12.7	1.32 [1.15–1.52]	1.19 [1.03–1.37]
		Yes	3443	664	19.3		
	Sedentary	No	4795	713	14.9	1.31 [1.14–1.51]	1.12 [0.97–1.30]
		Yes	1970	373	18.9		
	DM	No	5406	804	14.9	1.19 [1.02–1.39]	0.93 [0.79–1.10]
		Yes	1359	282	20.8		
	Smoking	No	5591	928	16.6	0.91 [0.75–1.10]	0.94 [0.79–1.15]
		Yes	1174	158	13.5		
TOD	PP > 60	No	5602	829	14.8	1.13 [0.94–1.36]	1.1 [0.92–1.32]
		Yes	1163	257	22.1		
	Left ventricular hypertrophy	No	6516	1022	15.7	1.44 [1.07–1.94]	1.36 [1.00–1.83]
		Yes	249	64	25.7		
	ABI < 0.9	No	6636	1060	16	1.14 [0.73–1.77]	1.06 [0.68–1.66]
		Yes	129	26	20.2		
History of cardiovascular disease	Stroke	No	6491	1030	15.9	1.10 [0.81–1.5]	1.00 [0.73–1.37]
		Yes	274	56	20.4		
	Ischemic heart disease	No	6277	996	15.9	0.96 [0.75–1.22]	0.89 [0.69–1.14]
		Yes	488	90	18.4		
	Atrial fibrillation	No	6386	973	15.2	1.76 [1.39–2.23]	1.53 [1.19–1.98]
		Yes	379	113	29.8		
	Heart failure	No	6582	1021	15.5	2.31 [1.68–3.18]	2.02 [1.44–2.83]
		Yes	183	65	35.5		
	Peripheral Artery Disease	No	6548	1045	16	0.98 [0.69–1.39]	0.88 [0.62–1.27]
		Yes	217	41	18.9		
Kidney disease	eGFR < 60 mL/min	No	6212	859	13.8	3.66 [3.01–4.46]	3.01 [2.40–3.78]
		Yes	548	226	41.2		
	Albuminuria	No	4500	687	15.3	1.70 [1.38–2.11]	1.43 [1.14–1.78]
		Yes	546	143	26.2		

OR = adjusted for sex, race, habitat, education level, employment, salary, and age; 95% CI = 95% confidence interval; aOR = adjusted for sex, race, habitat, education level, employment, salary, and age plus the variables included in its group: CVRF, TOD or CVD. CVRF: cardiovascular risk factors; HTN: hypertension; DM: diabetes mellitus; TOD: target organ damage; PP: pulse pressure; ABI: ankle-brachial index.

## Data Availability

Data sharing is not applicable to this study.

## Appendix A. The Investigators of the IBERICAN Study and of the Spanish Society of Primary Care Physicians (SEMERGEN) Foundation

Scientific Committee: Alfonso Barquilla García; Ángel Díaz Rodríguez; Carlos Escobar CervantesFrancisco Javier Alonso Moreno; Jesús Vergara Martín; Juan José Badimón; José Polo García; Luis Rodríguez Padial; Miguel Ángel Prieto; Rafael Vidal Pérez; Sergio Cinza Sanjurjo; Sonia Miravet Jiménez; Sonsoles Velilla Zancada, José Ramón Banegas, Vicente Martín Sánchez, Vicente Pallares Carratalá, Antonio Segura Fragoso y Rafael Manuel Micó Pérez.

*Andalusia*: Antonio López Téllez, Jesús Vergara Martin, María De Los Ángeles Ortega Osuna, Cristóbal Prieto Cid, Mª José Hidalgo Fajardo, José Lorente Serna, Ángel Domínguez Requena, Ricardo Alberola Cañizares, Manuel Ruiz Peña, Filomena Herrero Collado, Marcela Montes Vázquez, Rafael Ángel Carrascal Garrido, María Reyes Herrera Lozano, Beatriz Ortiz Oliva, Francisco José Anguita, Carmen Pérez Ibáñez, Carlos Alberto Cabrera Rodríguez, María José Cruz Rodríguez, Sandra Bonilla Ruiz, Rocío Reina González, Salome Abad Sánchez, Inmaculada Santana Martínez, Rafael Sánchez Jordán, Juan Mª Ramos Navas-Parejo, José Manuel Ramírez Torres, José María Beltrán Poveda, María Adoración De Cruz Benayas, Carmen Fernández Gil, Jon Iñaki Esturo Alcaine, Antonio Mora Quintero, Fernando Leiva Cepas, José Luis Carrasco Martín, Emilio García Criado, Mercedes Vázquez Blanco, Isabel Mora Ortiz, Leovigildo Ginel Mendoza, Juan Carlos Aguirre Rodríguez, Esperanza María Romero Rodríguez, José Acevedo Vázquez, Juan Gabriel García Ballesteros, María De La Paz Fernández Lara, Patricia Agüera Moreno, Eduardo Paños Maturana, Juan Manuel Ignacio Expósito, Noelia Carrillo Peñas, Carmen María Abad Faya, Ana Marina Almagro Duque, Rubén Torrescusa Camisón, Paloma Menéndez Polo, Marina Peña García, Cristina López Fernández, Ascensión Estepa Torres, Miguel Gutiérrez Jansen, Esperanza Loizaga González, Lisardo García Matarín, Enrique José Gamero De Luna, Javier Benítez Rivero, María José Gómez González, Carmen Gómez Montes, Juan Carlos Rodríguez Rodríguez, Juana María González Barranco, Josefa Ramírez Vizcaíno, María Ángeles Miranda Sánchez.

*Aragón*: Eva Trillo Calvo, Concepción Bayod Calvo, Susana Larripa De La Natividad, German Grasa Lambea, Emilio Jiménez Marín, Ana Cristina Navarro Gonzalvo, Antonio Pablo Martínez Barseló, Irene Peña León, Ángel González Pérez, Liliana Mahulea.

*Asturias:* María José Pérez Martínez, Ana Piera Carbonell, Margarita Alonso Fernández, María Montserrat Rueda Cuadrado, Rodrigo Abad Rodríguez, José Miguel Álvarez Cabo, Rubén Sánchez Rodríguez, Eva María Cano Cabo, Anny Romero Secin, Miguel Ángel Prieto Diaz, Juan Jesús García Fernández, Saul Suárez García.

*Balearic Islands*: Fernando García Romanos, Antonia Moreno González, María Lara Amengual Sastre, Susana Martínez Palli, José Alfonso Ramón Bauza, Jose Ortiz Bolinches, Carmen Fernández Fernández, María Isabel Orlandis Vázquez, Ana Sanchis Mezquita, Fernando Unceta Aramburu, Juan Fernando Peiró Morant, Ana Moyá Amengual, Mateu Seguí-Díaz. Basque Country: José Félix Zuazagoitia Nubla, Ana Echevarría Ituiño, Gregorio Mediavilla Tris, María Carmen Noriega Bosch, Esther González, María Luisa Ruiz Macho, Ruth Sendino Del Olmo, Asunción Olagorta De Prado, Ana López De Viñaspre Muguerza, Jesús Iturralde Iriso, Mª Rosario Virtus Iñurrieta, Lucas Ulloa Bahamonde.

*Canary Islands:* Isidro Godoy García, Fernando Rubio Sevillano, María Isabel González González, Marta Pérez Souto, Raquel De León Contreras, Sara Isabel Almeida González, Irene Almería Diez, Virginia María Mirabal Sánchez, Francisco Jose Escobar Lavado, Yoel Anta Pérez, Nayra Sánchez Hernández, Juan Luis Alonso Jerez, Ricardo Koch, Nayra Ramírez Mendoza, Héctor Suárez Hernández, Francisco Jesús Morales Escobar.

*Cantabria:* E. Lidia Gutiérrez Fernández, Fernando Andrés Mantecón, Ana Belén García Garrido, Asunción Vélez Escalante, Luisa Alonso Rentería, Jesús Sainz Jiménez, Guillermo Pombo Alles, Esperanza Rueda Alonso.

*Castilla La Mancha:* Juan Antonio Divisón Garrote, Pedro Martínez Sotodosos, Juan Antonio Vivancos Fuster, María García Palencia, José Ambrosio Torres Moraleda, Sara González Ballesteros, Ana Carmen Gil Adrados, Antonio González Cabrera, Miguel Ángel Babiano Fernández, Guillermo Rico García, Juan José Criado-Alvarez, Pilar Torres Moreno, Francisco Javier Arribas Aguirregaviria, Alicia Sahuquillo Martínez, Lourdes María Santos Bejar, Miguel Laborda Peralta, Raúl Piedra Castro, Carlos Santos Altozano, Lucia González-Tarrio Polo, Pedro Valiente Maresca, Reinilda Mota Santana, Noemi Elizabeth Terrero Ledesma, Noelia Garrido Espada, Francisco Javier Alonso Moreno, Gabriela Delia Rosa Zambrana Calvi, Cristina de Castro Mesa, Blanca Cordero García, Pilar Sorrius Sitges, Ana María de Santiago Nocito, César Lozano Suárez.

*Castilla y León:* Juan Lorenzo Gutiérrez Montero, Juan Ignacio López Gil, María Dolores Fernández Ortega, Miren Elizari Roncal, María Ascensión López Serrano, Nuria Esther Adrián De La Fuente, Belén Angulo Fdez. De Larrea, Naiara Cubelos Fernández, Guiomar Luz Ferreiro Gómez, Diana Gómez Rodríguez, Sonia María Andrés Tuñón, María Ajenjo González, Serafín De Abajo Olea, Juan José León Regueras, César Manuel Gallego Nieto, Delio Vázquez Mallada, María De La O Gutiérrez García, Pablo Baz Rodríguez, José Ignacio Ferradal García, Blanca Delia De Román Martínez, Ana Arconada Pérez, Omar Mahmoud Atoui, Álvaro Morán Bayón, María Teresa Armenteros Del Olmo, Francisco Javier García-Norro Herreros, Enrique Méndez Rodríguez, Diana María Narganes Pineda, Ángel Díaz Rodríguez, Verónica Ortiz Ainaga, Milagros Sonlei Sánchez Guevara, Laura Villota Ferreiro, M Teresa Grande Grande, Francisco Vicente Martínez Gracia, Jesús Palomo del Arco.

*Catalonia:* María Dolores Moriano García, Beatriz Jiménez Muñoz, Gemma Rovira Marcelino, Diana Elizabeth Fernández Valverde, Roser Rodó Bernadó, María Teresa Ortiz Lupiañez, Najlaa Najih, José María Diéguez Parra, Mª Rosa Benedicto Acebo, Mari Luz Bravo Vicien, Alberto Ramón León Estella, Juan Antonio Muñoz Gómez, Alicia Mostazo Muntané, Isabel Ortega Abarca, Anna Gasol Fargas, Brenda Elizabeth Riesgo Escudero, Susana Elizabeth Riesgo, Edgar Zaballos Castellvi, Celia Cols Sagarra, Marta Herranz Fernández, Josep Alins Presas, Idaira Damas Pérez, Rosa M Alcolea García, Ines Monte Collado, Roberto Genique Martínez, María José Guasch Villanueva, Sònia Miravet Jiménez, Teresa Rama Martínez, Lucio Pinto Pena, Josefa María Panisello Royo, Inés Gil Gil, Carlos Gómez Ruiz, Rita Sahun Font, Anna Fuentes Lloveras.

*Community of Madrid:* Alberto Calderón Montero, María Del Mar Zamora Gómez, Elena Alarcón Cebrián, Mª José Piñero Acin, Celia Pecharroman Sacristán, M Soledad Mayayo Vicente, Mª Paz Pérez Unanua, Nuria Marañón Henrich, Saray Gómez Monreal, Sonia Redondo De Pedro, Blanca Sanz Pozo, Irene Moreno Martínez, Beatriz López Uriarte, Carmelina Sanz Velasco, Amaya Gárriz Aguirre, Montserrat Rivera Teijido, German Reviriego Jaén, José Ignacio Aza Pascual-Salcedo, Josefa Vázquez Gallego, Julia Caballer Rodilla, Aida Herrera, Ezequiel Arranz Martínez, Ana María Gómez Calvo, Paula Morán Oliva, Mª Milagros González Béjar, Julio Antonio Heras Hitos, Olga Garcia Vallejo, Manuel De Jesús Frías Vargas, María Jesús Castillejo Boguerin, Aurora García Lerín, Miguel Ángel María Tablado, Elena Concepción García García, Leticia De Miguel Acero, Carmen Zárate Oñate, Aránzazu Barranco Apoita, María Ester Montes Belloso, Ana Maria Huertas Velasco, Rafael Sáez Jiménez, Julia Natividad García Pascual, María Clemencia Zuluaga Zuluaga, Mª Cruz Díez Pérez, Antonio Ruiz García, Cristina Murillo Jelsbak, Virginia Lasso Oria, Amelia González Gamarra, Elena Rodilla Rodilla, Alberto Galgo Nafría, María Mestre de Juan, Mª Carmen García Albiñana, Mª del Pilar Moreno Cano, Paula Hernanz López, Paloma Casado Pérez.

*Extremadura:* Jacinto Espinosa García, José Ignacio Prieto Romo, Leandro Fernández Fernández, Javier Sierratapia, Nieves Moreno Regidor, Francisco Javier Zaballos Sánchez, Ana Moreno Moreno, Francisco Carramiñana Barrera, Juan José Torres Vázquez, María José Gamero Samino, Miguel Ángel De Santiago Rodríguez, Pablo Rafael Gómez Martínez, Antonio Carlos Elías Becerra, Javier Soto Olivera, Víctor Cambero, Julián Domínguez Ávila, Andrés Simón Fuentes, Jorge Manuel De Nicolás Jiménez, Dimas Igual Fraile, Guadalupe Nieto Barco, Ignacio Araujo Ramos, Mª Luz Serrano Berrocal, Francisco Buitrago Ramírez, Minerva Gallego Marcos, Félix Suarez González, Victoriano Chavero Carrasco, José Polo García, Francisco Guerra Peguero, Francisco Javier Sánchez Vega, Manuel Tejero Mas, Alba Palmerín Donoso, Miguel Turégano Yedro, María Beatriz Esteban Rojas, Fátima Cabezudo Moreno, Nawson Elver Quevedo Saldaña, María Del Mar García Fenés, Alfonso Barquilla García, Timotea Garrote Florencio, José María Fernández Toro, Vicente Caballero Pajares, María José Gómez Barquero.

*Galicia:* Alejandra Rey Rañal, Elena García Del Río, Enrique Nieto Pol, Julio Álvarez Fernández, Pilar Alonso Álvarez, Mª Luisa Jorge Gómez, Antonio Calvo Guerrero, Isabel Celemín Colomina, Lucia Barreiro Casal, Juana Fernández Moreno, Mª Angelines Carballal Martínez, Nabor Díaz Rodríguez, Carlos Moral Paredes, Dolores Recarey García, Francisco Javier Iglesias Mato, Antonio Fouz Ulloa, Amparo Fidalgo González, Noelia Dios Parada, Patricia Conde Sabarís, Ana Isabel Rodríguez Pérez, Ana Inés García Palacio, Víctor Julio Quesada Varela, Lidia Romero Iglesias, Ángel Lado Llerena, Carmen Lires Rodríguez, María Luisa Carretero Díaz, José Carreira Arias, José Luís Vázquez Camino, María Del Carmen Torreiro Penas, Sandra Yáñez Freire, Sergio Cinza Sanjurjo, Daniel Rey Aldana, Carlos Piñeiro Díaz, Portal González Lorenzo, José Rodríguez Campos, Rubén Blanco Rodríguez, Manuel Portela Romero, Lucía Vilela de Castro.

*La Rioja*: Sonsoles María Velilla Zancada, Rafael Crespo Sabarís, Oscar Fernando Isaula Jiménez. Melilla: Jesús Manuel Gonzalez Puga, Jorge Antonio Benaín Ávila, Óscar Del Toro González. Murcia: Vicente Llorca Bueno, Ana María Ballesteros Pérez, Domingo J. Rubira López, Mª Dolores Esteve Franco, Elena Sánchez Pablo, María Teresa Palacios López, Juan Castillo Meroño, José María Lobo Martínez, Isabel María Peral Martinez, J. Eduardo Carrasco Carrasco, Armando Santo González, Juan Gomáriz García, Beatriz Ríos Morata.

*Navarra:* Laura Sánchez Iñigo, Inés Sanz Pérez.

*Valencian Community*: Vicente Pascual Fuster, Mª Dolores Aicart Bort, Natividad Vázquez Gómez, Carlos Lluna Gasco, Teresa Amorós Barber, Pedro Antonio Medina Cano, Miguel Monteagudo Moncho, Mª Jesús Larré Muñoz, Raquel Navarro Hernández, Francisco José Martínez Egea, Antonio Tramontano, Marta Ferrer Royo, Belén Persiva Saura, Juan A. Contreras Torres, José Mª Tirado Moliner, Alejandro Salanova Penalba, Ariadna Cucó Alberola, Fernando María Navarro I Ros, Enrique Beltrán Llicer, Ana Seoane Novás, Inmaculada Martín Valls, Gracia Verdú Mahiques, Enrique Peña Forcada, Nieves Aguilar Gómez, Francisco Javier Sanz García, M Dolores Paradís Bueso, María Eugenia Alegre Romero, Antonio Francés Camus, María Amparo Anton Peinado, Rosa Latorre Santos, Mª Asunción Palomar Marín, María Carmen Botella García, Eva Sánchez Fresquet, Pedro Sala Paños, Tomás Sánchez Ruiz, Rosa Ana Valero Valero, María Seoane Vicente, Magdalena Martin Llinares, Antonio Masiá Alegre, José Luis Llisterri Caro, Irene Lluch Verdu, Vicente Pallarés-Carratalá, Francisco Valls Roca, Rafael Manuel Micó Pérez, Carmen Barceló Dupuy, Elena Benages Vicente, María José Gimeno Tortajada, Mercedes Calleja del Ser, Martín Menéndez Rodríguez, Rosalía Victoria Carbonell Castelló.
